# Clinical outcomes of a digitally delivered structured lifestyle program in type 2 diabetes: a randomized controlled trial from India

**DOI:** 10.3389/fendo.2026.1920317

**Published:** 2026-08-18

**Authors:** Chhavi Mehra, Annie Mattilda Raymond, Banshi Saboo, Aravinda Jagadeesha, Karthik S. M., Mudit Sabharwal, Karunesh Kumar, Ankitha Sequeira, Jeny Joseline J., Shivtosh Kumar

**Affiliations:** 1 Research and Development, Ragus Healthcare Private Limited, Bengaluru, India; 2 Dia Care Diabetes Hormone Clinic, Ahmedabad, India; 3 Dr. Aravind’s Diabetes Centre, Bengaluru, India; 4 Narayana Hospital, Basant Health Center, Bengaluru, India; 5 Dharma Diabetes and Metabolic Clinic, Delhi, India; 6 Diabliss Medical Center, Bengaluru, India

**Keywords:** behavior change, digital health, glycemic control, lifestyle intervention, precision nutrition, type 2 diabetes

## Abstract

**Background:**

Effective type 2 diabetes (T2D) management extends beyond pharmacotherapy to include structured lifestyle interventions and continuous digital support. Accordingly, this study aimed to evaluate the effectiveness of an integrated, multicomponent 90-day healthcoach-assisted lifestyle intervention program in improving glycemic and related metabolic outcomes among patients with T2D, with assessment of benefits across adherence levels.

**Methods:**

This prospective, multicenter randomized controlled trial (RCT) included 149 participants with T2D allocated to intervention (n=87) and control (n=62) groups, with subgroup stratification of intervention participants into high-adherence (≥35%; n=62) and low-adherence (<35%; n=25) groups based on median composite adherence scores. The intervention group received a structured lifestyle intervention on diet, exercise, and mental wellness alongside standard of care (SOC), while controls received SOC alone for 90 days. Glycemic, anthropometric, and diabetes distress outcomes were compared between groups and across adherence subgroups. Statistical significance was assessed for all outcomes.

**Results:**

The intervention group demonstrated statistically significantly greater reductions (p<0.001) compared with the control group in glycated hemoglobin (HbA1c) (1.46 ± 1.82 vs. -0.07 ± 1.22%), fasting blood sugar (FBS) (18.83 ± 60.46 vs. -10.84 ± 55.20 mg/dL), body mass index (BMI) (0.55 ± 4.32 vs. 0.18 ± 0.78 kg/m^2^), body weight (1.49 ± 2.22 vs. 0.26 ± 2.57 kg), and waist circumference (1.86 ± 2.53 vs. -0.09 ± 3.93 cm). Homeostatic model assessment for insulin resistance (HOMA-IR) decreased (0.36 ± 6.63 vs. -0.27 ± 4.58), while HOMA of β-cell function (HOMA-B) improved (36.28 ± 134.32 vs. 8.72 ± 79.1), as compared with the control group. Further, diabetes-related distress significantly improved in the intervention group, shifting from moderate to low/no distress, whereas the control group approached the threshold for moderate distress (0.57 ± 0.77 vs. 0.27 ± 0.71). Participants with high-adherence showed significant reductions in HbA1c (1.7 ± 1.5 vs. 0.8 ± 1.3%), FBS (25.2 ± 60 vs. 3.1 ± 59.9 mg/dL), and weight (1.6 ± 2.3 vs. 1.4 ± 2.1 kg) compared to low-adherence participants.

**Conclusion:**

This digitally delivered lifestyle intervention program produced clinically meaningful improvements in glycemic control, anthropometric measures, and diabetes related distress compared with standard care alone, with greater benefit among high adherence participants. These findings support integrating structured, coach guided digital lifestyle programs into routine T2D care, particularly in resource limited, real-world settings such as India.

**Clinical trial registration:**

https://ctri.nic.in/Clinicaltrials/pmaindet2.php?EncHid=MTA2NDAx&Enc=&userName=, identifier CTRI/2024/05/068043.

## 1 Introduction

Diabetes mellitus constitutes a substantial and rapidly escalating global health burden, with the adult diabetes population increasing from 151 million in 2000 to 588.7 million in 2024 and projected to reach 852.5 million by 2050. This growing burden is particularly evident in low- and middle-income countries (LMICs), including India, which accounts for one of the largest diabetes populations worldwide, with cases rising from 32.7 million in 2000 to 89.8 million in 2024 and projected to reach 156.7 million by 2050 (1). The increasing prevalence of T2D has been largely driven by rapid urbanization, aging populations, sedentary lifestyles, and unhealthy dietary habits, contributing substantially to obesity, insulin resistance, and metabolic dysfunction. Beyond prevalence alone, diabetes imposes considerable morbidity, mortality, psychological burden, and healthcare costs, thereby emphasizing the importance of effective glycemic control and long-term management strategies for individuals with T2D (2).

In addressing the need for effective long-term diabetes management, it is increasingly evident that pharmacological regimens alone are often insufficient to reverse the underlying metabolic dysfunction associated with T2D (3). Real-world evidence from India demonstrated that 76.6% of patients with T2D failed to achieve recommended glycemic targets despite ongoing mono- and combination antidiabetic therapy, highlighting persistent challenges in long-term glycemic management (4). Furthermore, diabetes clinic-based evidence suggests that increasing disease duration often necessitates escalation from monotherapy to dual, triple, and insulin-based regimens to achieve glycemic targets, underscoring the progressive difficulty of sustaining long-term glycemic control through pharmacotherapy alone (5).

Given the multifactorial and heterogeneous nature of T2D, unhealthy dietary patterns with nutritional inadequacies, physical inactivity, and psychological health collectively contribute to adverse glycemic outcomes, increased risk of comorbid depression, and reduced adherence to prescribed therapy (6). Accordingly, international and Indian diabetes guidelines, including the American Diabetes Association/European Association for the Study of Diabetes (ADA/EASD) consensus report and the RSSDI clinical practice guidelines, recommend a holistic, patient-centered approach that integrates medical nutrition therapy, structured physical activity, psychological support, weight management, and pharmacotherapy to achieve optimal glycemic control and reduce long-term complications (7, 8).

The Diabetes Prevention Program demonstrated that intensive lifestyle intervention reduced T2D progression by 58% among high-risk cohorts (9). In addition, even modest weight reduction of more than 5% has been shown to improve HbA1c, lipid profile, and blood pressure among individuals with T2D (10). Emerging evidence suggests that even when T2D remission is achieved, continued adherence to lifestyle therapy remains essential for maintaining overall metabolic health (11). Extending this evidence, intensive lifestyle programs that incorporate continuous engagement and personalized guidance have demonstrated superior efficacy in improving glycemic control and sustaining long-term behavioral adherence compared with passive, information-only approaches (12).

Despite the established benefits of lifestyle-based interventions, sustaining long-term adherence to diabetes self-management remains challenging due to the longitudinal and behavior-dependent nature of lifestyle modification. Evidence from an Indian clinical setting reported medication adherence, self-care practices, and environmental as substantial barriers, highlighting persistent challenges in translating guideline-based recommendations into sustained real-world practice (13).

To address these challenges, advances in digital technologies have emerged as promising strategies for delivering lifestyle consultations through mobile health platforms, app-based coaching programs, and remote self-management interventions. Evidence from systematic reviews and meta-analyses indicates that digital interventions can improve glycemic control, engagement, and adherence in T2D, particularly when combined with personalized coaching and continuous professional support (14). These gains are further strengthened by behavioral support strategies, such as health coaching, mobile-phone counselling, and goal-setting, which improve adherence to dietary and physical activity regimens (15). Similarly, multifaceted lifestyle interventions integrating nutrition therapy, physical activity, and psychological support have demonstrated benefits for adherence, metabolic outcomes, and overall well-being (16).

Despite these encouraging findings, most evidence on digital diabetes interventions originates from Western populations, limiting its direct applicability to the Indian context due to differences in healthcare access, sociocultural factors, digital literacy, and lifestyle practices As emerging studies from India have explored mobile-based counselling, telephonic follow-up, and short-term digital lifestyle programs, evidence regarding comprehensive multicomponent digital interventions integrating nutrition, physical activity, behavioral coaching, and continuous two-way support in real-world diabetes care settings remains limited.

To address this gap, the present RCT evaluated the efficacy of an interactive digital lifestyle intervention to improve glycemic and anthropometric outcomes among individuals with T2D over 90 days in a real-world diabetes care setting. Crucially, to our knowledge, this study represents one of the earliest Indian real-world trials to evaluate a multicomponent digital lifestyle intervention, encompassing structured dietary guidance, coach-led behavioral support, and continuous remote monitoring, as an adjunct to standard pharmacological care within a specialty clinic population, with outcomes further systematically stratified by program adherence.

## 2 Methodology

### 2.1 Study design

**Figure 1:**
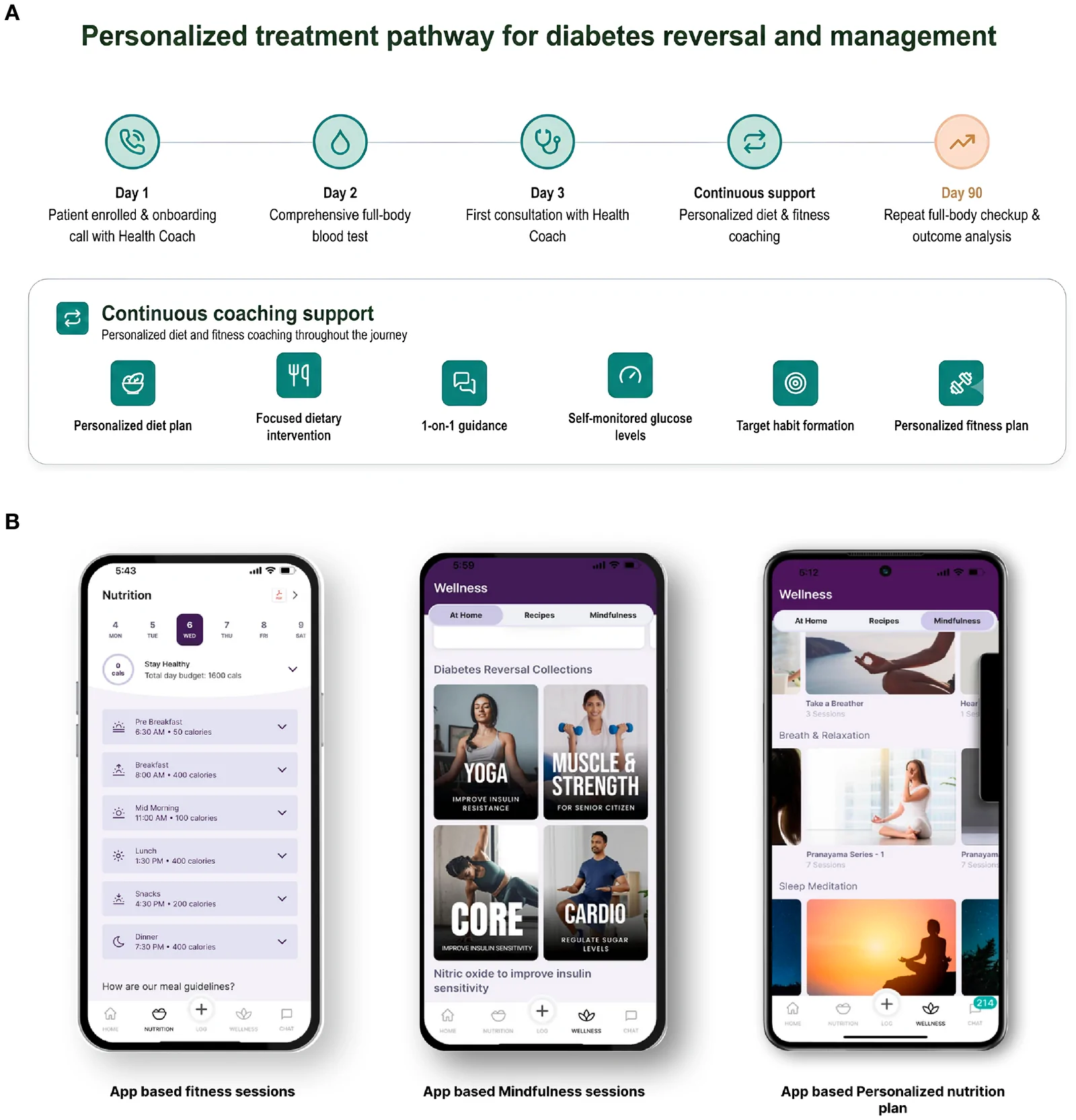
**(a)** Illustration of overall participant flow from enrollment through randomization, intervention delivery, follow-up, and final outcome analysis. **(b)** Mobile application interface. Representative screenshots illustrating key intervention delivery modules.

This study was a prospective, multicenter, randomized controlled trial conducted to evaluate the effectiveness of a comprehensive multicomponent digital lifestyle intervention delivered in addition to standard of care (SOC) compared with SOC alone among individuals with T2D. The overall participant flow from enrollment through randomization, intervention delivery, follow-up, and final outcome analysis is illustrated in **Figure 1a**.

The intervention was implemented under real-world diabetes care conditions over a 90-day study period. The primary objective was to evaluate the effect of the intervention on glycemic control and anthropometric outcomes, assessed by changes in glycated hemoglobin (HbA1c), fasting blood glucose (FBS), body weight, and body mass index (BMI). Secondary objectives included evaluation of waist circumference, insulin resistance and β-cell function indices (HOMA-IR and HOMA-B), and diabetes-related distress measured using the Diabetes Distress Scale (DDS-17). The 90-day follow-up period was selected to capture clinically meaningful changes in metabolic, anthropometric, and psychological outcomes.

### 2.2 Recruitment and study setting

The RCT was conducted between July 2024 and June 2025. Participants (n=237) were recruited from four specialized diabetes care centers in India: Dia Care-Diabetes Hormone Clinic (Ahmedabad), Dharma Diabetes and Metabolic Clinics (New Delhi), Aravinda Diabetic Centre (Bangalore), and Diabliss Medical Center (Bangalore). Individuals attending these centers were screened for eligibility based on predefined inclusion and exclusion criteria. A total of 237 individuals were screened for eligibility based on predefined inclusion and exclusion criteria. Of these, 180 eligible participants provided written informed consent and were subsequently randomized into the study.

### 2.3 Participant eligibility screening and informed consent

Eligible participants were adults aged 18 years or older with a confirmed diagnosis of T2D and baseline HbA1c ≥7.0%, who were willing to comply with the study protocol and provided written informed consent prior to enrollment. Participants were excluded if they had participated in another clinical trial within the preceding 30 days, were pregnant or lactating, or had alternative or overlapping diabetes diagnoses, including type 1 diabetes, gestational diabetes, or diabetic ketoacidosis. Additional exclusion criteria included severe comorbid conditions such as major cardiovascular disease, oncological disorders, renal disorders including end-stage renal disease, or significant renal impairment (creatinine clearance <30 mL/min). Prior to enrollment, all participants were informed regarding the study objectives, intervention procedures, outcome assessments, potential benefits, confidentiality measures, and their right to withdraw from the study at any stage without affecting routine clinical care.

### 2.4 Ethical considerations, trial registration, and reporting standards

The clinical trial protocol and all participant-related documents were reviewed and approved independently by the Institutional Ethics Committee of Aatman Hospital, Ahmedabad; the ACE Independent Ethics Committee, Bangalore; and the Good Society for Ethical Research Institutional Ethics Committee (GSER-IEC), New Delhi (Study Code: MSPL/SDR/001/2024). This study was conducted in strict accordance with the Declaration of Helsinki, the Indian Council of Medical Research (ICMR) ethical guidelines (2006), and the International Council for Harmonisation Good Clinical Practice (ICH E6-GCP) standards. Prior to enrollment, written informed consent was obtained from all participants. Throughout the duration of the study, dedicated clinical trial coordinators actively monitored participant safety and protocol adherence.

Following these ethical approvals and prior to participant recruitment, the trial was officially registered with the Clinical Trials Registry–India on 29 May 2024 (Registration Number: CTRI/2024/05/068043). The full, publicly available registry record and trial parameters can be accessed at: https://ctri.nic.in/Clinicaltrials/pmaindet2.php?EncHid=MTA2NDAx&Enc=&userName=.

To ensure transparency and rigor in communicating the study’s outcomes, the trial was designed, conducted, and reported in accordance with the Consolidated Standards of Reporting Trials (CONSORT 2010) statement (17). Furthermore, given the technology-assisted nature of the intervention, relevant recommendations from the CONSORT-EHEALTH guidelines (18) were integrated into the framework. This methodology ensures comprehensive, transparent reporting regarding the functionality of the mobile application, digital engagement features, health coach involvement, adherence monitoring, and participant support mechanisms.

### 2.5 Randomization

Following eligibility screening and informed consent, participants were randomized in a 1:1 ratio to the intervention or control group. The randomization sequence was generated centrally by the study’s contract research organization (CRO), which prepared a pre-specified set of allocation cue cards (chits) corresponding to a 1:1 intervention-to-control ratio. This sequence was distributed electronically (as a PDF) to each site’s clinical research coordinator (CRC), who printed and shuffled the chits locally prior to each enrollment session.

Allocation concealment was maintained by having the site CRC rather than the Principal Investigator (PI), prepare and present the shuffled, identical-appearing chits at the time of enrollment. Following eligibility confirmation and informed consent, each participant personally selected a chit at random from the shuffled set; the PI remained unaware of the pending allocation and had no role in generating, shuffling, or selecting the chit. This process ensured that neither the enrolling PI nor the participant could predict or influence group assignment prior to selection.

Given the behavioral nature of the intervention, participants and coaching staff could not be blinded to group assignment following allocation. However, outcome assessment was conducted independently of the intervention delivery team: laboratory-based glycemic parameters (HbA1c, FBS) were processed by laboratory technicians blinded to group allocation, and anthropometric measurements (body composition analysis) were captured via on-site body composition analysis with automated, blinded electronic data recording. DDS responses were self-reported by participants and recorded by research coordinators via structured case report forms. Health coaches had no role in outcome data collection and accessed outcome reports only for counselling purposes after data capture.

SOC across all four participating centers reflected routine clinical practice as delivered by treating physicians with established expertise in diabetes management, generally guided by the Research Society for the Study of Diabetes in India (RSSDI) and, where relevant, the American Diabetes Association (ADA) recommendations. No standardized SOC protocol, checklist, or structured lifestyle-counseling mandate was imposed across sites, consistent with real-world clinical practice in India. While physicians could refer patients to an in-house or consulting nutritionist for lifestyle counseling, such referrals were discretionary rather than mandated, and uptake depended on patient-borne out-of-pocket costs, as structured nutrition or lifestyle counseling is not typically covered under health insurance in this setting. Across all four sites, routine follow-up visit frequency was consistent at approximately once every three months, with no scheduled contact between visits outside of clinical emergencies. This reflects the customary standard of care delivery model across Indian diabetes clinics generally, rather than a site-specific practice difference, and was therefore not independently harmonized beyond this shared underlying pattern of care.

### 2.6 Interventions

Participants assigned to the intervention group received a comprehensive, technology-assisted lifestyle modification program for 90 days in addition to standard of care (SOC). The intervention consisted of an integrated, multicomponent lifestyle program with structured macronutrient targets, prescribed physical activity, behavioral and mental wellness coaching, regular follow-up consultations, self-monitoring support, and continuous digital engagement delivered by dedicated healthcare professionals, including nutritionists, fitness coaches, and diabetes educators.

In contrast, participants allocated to the control group received SOC alone throughout the study duration. SOC included routine diabetes pharmacotherapy, physician consultations, laboratory monitoring, and general lifestyle advice in accordance with standard clinical practice guidelines. Participants in the control group additionally received routine diabetes management and follow-up provided in accordance with standard clinical practice guidelines, and a single baseline lifestyle counseling session; however, they did not receive structured digital lifestyle intervention, customized coaching, continuous monitoring, or app-based follow-up support.

During the initial two weeks of the 90-day intervention period, participants in the intervention group were introduced to a limited set of intervention components selected according to individual preferences, readiness for adherence, and physiological profile. This phased implementation approach was intended to facilitate gradual adaptation, improve participant engagement, and enhance the feasibility of sustained long-term adherence to the lifestyle intervention.

Throughout the intervention period, participant adherence and clinical progress were continuously monitored using the Sugar.Fit mobile application (Version 9.0). The platform enabled remote tracking, regular feedback delivery, and direct communication between participants and healthcare coaches. Continuous monitoring further allowed periodic review and refinement of lifestyle recommendations based on glycemic trends, anthropometric changes, and participant-reported feedback. A schematic overview of the intervention framework and digital care pathway is presented in **Figure 1b**.

The intervention framework comprised three core domains: nutritional therapy, structured physical activity, and psychological/behavioral wellness support.

### 2.7 Nutritional intervention

Dietary modification served as the core therapeutic component of the Sugarfit’s Diabetes Reversal and Management Program (SDRMP), designed to improve glycemic control, support weight management, and foster long-term behavioral adherence. To establish a baseline, participants underwent a comprehensive dietary assessment that evaluated routine eating patterns, meal timing, cultural practices, and specific weight goals. This evaluation combined 24-hour dietary recalls covering both weekdays and weekends with a structured food frequency questionnaire developed for program use to quantify the habitual intake of major food groups, processed foods, and sugar-sweetened beverages.

The data gathered from this assessment directly informed targeted nutritional prescriptions using a structured energy-balance approach. Total Daily Energy Expenditure (TDEE) was determined by calculating the participant’s Basal Metabolic Rate (BMR) and multiplying it for physical activity level (PAL), thereby establishing a physiological reference for energy estimation. Based on this reference, dietary prescriptions were aligned with recommended dietary intake guidelines and customized to support participants’ specific weight-management goals. This calculation allowed health coaches to establish a precise metabolic baseline, from which specific caloric targets were assigned according to the participant’s BMI and clinical objectives.

Energy restriction was implemented for individuals with overweight (BMI: 23.0-24.9 kg/m^2^), obese (BMI: ≥25.0 kg/m^2^), or high-risk obese (BMI: ≥27.5 kg/m^2^), additional caloric intake was prescribed for those who were underweight (BMI: <18.5 kg/m^2^), and energy intake was matched to estimated requirements for participants with normal weight (BMI: 18.5-22.9 kg/m^2^), according to Asian Indian-specific BMI classification guidelines (19), consistent with subsequent expert consensus updates (20).

Macronutrient distribution followed a structured dietary guidance framework consistent with contemporary nutrition therapy recommendations (7, 8), which favor flexible macronutrient ranges over fixed prescriptive ratios. Prescribed distributions typically fell within 15–20% protein (prioritizing lean sources), 25–30% fat (favoring mono- and polyunsaturated fatty acids while limiting saturated and trans fats), and 45–55% carbohydrate (emphasizing fiber-rich complex carbohydrates while minimizing refined sugars and ultra-processed foods). Grain-free dietary patterns were introduced on a discretionary basis when clinically indicated by specific metabolic profiles or tolerability requirements.

To facilitate real-world compliance, participants were provided with structured 7-day meal plans calibrated to their specific caloric targets and cultural preferences. This practical approach was reinforced through clinical counseling focused on portion control, mindful eating, and meal preparation strategies. To minimize recall bias and ensure data accuracy, participants transitioned to real-time digital dietary monitoring. They maintained electronic food logs and uploaded meal photographs, which allowed trained health coaches and nutrition specialists to validate actual intake, verify portion sizes, and identify deviations from the prescribed plan.

Additionally, time-restricted eating (TRE) and chrononutritional protocols were implemented as mandatory components of the nutritional regimen for all participants. Under this framework, participants were instructed to maintain consistent meal schedules, reduce snacking frequency, and complete dinner in the early evening, preferably by 07:30 PM, while enforcing a definitive cessation of caloric intake at least three hours before bedtime. Adherence to these daily feeding schedules initiated with a 12-hour overnight fasting window. As the intervention progressed, this fasting window was extended up to a maximum of 14 hours for individual participants based on their demonstrated adherence and glycemic response. This progressive, individualized escalation reflected the need to address the sustainability challenges often associated with rigid, uniform fasting protocols in real-world clinical settings. Accordingly, extensions were introduced gradually, guided by behavioral tolerance, and only when clinically appropriate, consistent with the program’s broader adaptive coaching framework (Section 2.10.3), to maximize long-term adherence and clinical efficacy rather than impose a fixed regimen.

### 2.8 Physical activity intervention

To complement the nutritional interventions, structured exercise regimens were developed for each participant. To foster sustainable behavioral adaptation and minimize sedentary behavior, initial prescriptions prioritized gradual increases in physical activity over strenuous activity. Exercise programming was calibrated based on baseline functional capacity, mobility constraints, medical comorbidities, occupational demands, and individual metabolic goals.

During the intake evaluation, baseline physical activity levels (PAL) were quantified and categorized as sedentary/lightly active (PAL 1.40-1.69), moderately active (PAL 1.70-1.99), vigorously active (PAL 2.00-2.40), or extremely active (PAL >2.40) as defined by the last Food and Agriculture Organization/World Health (FAO/WHO/UNU) expert consultation on human energy requirements (21). These classifications, alongside occupational habits, guided the initial prescription and subsequent progression metrics. For participants with predominantly sedentary occupations, daily routines were modified to incorporate structured movement breaks, stretching, and desk-based activities. Step-count goals were assigned to optimize non-exercise activity thermogenesis, with hourly targets to interrupt prolonged sitting. For individuals with frequent travel commitments or restricted facility access, adaptable, equipment-free modalities such as soleus push-ups, were prescribed to maintain training continuity.

The core exercise framework comprised a combination of brisk walking, aerobic conditioning, and resistance training. Progression of both intensity and volume was titrated according to individual physiological tolerance and functional improvement. While gym-based workouts were utilized when feasible, broad accessibility was maintained by offering four live, virtual fitness sessions daily across varying intensity tiers. Additionally, an on-demand digital library of recorded sessions (e.g., yoga, HRX workouts, core-conditioning routines, strength-training sessions, and mobility exercises) ranging from 15 to 40 minutes provided scheduling flexibility for participants unable to attend live classes.

To optimize metabolic health and improve glycemic management, resistance and strength-training and moderate-intensity aerobic exercises were actively encouraged during morning hours; when clinically appropriate and well-tolerated, these sessions were performed in a fasted state prior to the first meal of the day. To specifically target postprandial glucose volatility, low-intensity postprandial walking for 15–30 minutes following major meals was a mandatory component of the protocol.

Activity adherence and performance metrics, including daily step count, and exercise duration, were continuously monitored using inbuilt wearable fitness trackers synchronized with the Sugar.Fit mobile application. Data from connected devices were automatically integrated into the platform, while app engagement metrics were simultaneously tracked to assess participant adherence and interaction with the intervention. These objective datasets allowed health coaches to periodically review and refine exercise prescriptions as the participant’s functional capacity evolved. To mitigate training monotony and sustain long-term compliance, participants maintained access to a diverse matrix of modalities, including instructor-led classes, and continuous clinical coaching support.

### 2.9 Mental wellness intervention

Psychological support was embedded within the broader lifestyle intervention framework to enhance long-term adherence and strengthen self-efficacy for sustained behavioral change. This integrated behavioral and psychological care was delivered through a dual-level model involving psychologists and trained health coaches, wherein psychologists conducted structured virtual consultations while health coaches reinforced stress-management strategies during routine coaching interactions. To ensure standardized delivery of care, health coaches underwent structured psychologist-led training to strengthen their capacity for integrated mental health support and empathetic communication, enabling consistent application of behavioral strategies across participant interactions.

Accordingly, diabetes-related distress was assessed using the widely accepted DDS-17 and used to guide individualized psychological support (22). Participants identified as experiencing high diabetes distress (mean DDS score ≥3.0) at baseline were referred for structured 60-minute virtual consultations delivered by qualified psychologists. These sessions focused on evidence-based coping strategies, emotional regulation, stress reduction, problem-solving skills, and strengthening self-management behaviors. Participants with low-to-moderate distress (mean DDS score <3.0) at baseline received ongoing behavioral support from trained health coaches, who reinforced stress-management strategies alongside dietary and physical activity recommendations during routine coaching interactions.

Additionally, recognizing the interrelationship between psychological well-being and lifestyle adherence, structured stress-management practices were incorporated into the program. These components were delivered through 60-minute live instructor-led group sessions supplemented by app-based digital modules, including guided meditation, breathing exercises, mindfulness techniques, and Yoga Nidra, enabling flexible participation and sustained engagement. These interventions were designed to reduce perceived stress, enhance overall well-being, and reinforce adherence to lifestyle modifications.

### 2.10 Behavioral and coaching support

Behavioral support was delivered through a digitally enabled health coaching system designed to facilitate lifestyle modification, continuous engagement, and adherence to the intervention protocol. It integrated digital self-monitoring, individualized coaching, educational content, and adaptive feedback mechanisms to promote sustained behavioral change throughout the 90-day intervention period.

#### 2.10.1 Digital coaching platform

Participants utilized the digital app for intervention delivery, engaging with the platform and monitoring their progress by recording dietary intake through food-image submissions, logging self-monitored blood glucose readings, tracking body weight and waist circumference measurements, and synchronizing wearable-derived physical activity data including step count, distance travelled, and active energy expenditure. The platform also provided access to educational materials related to nutrition, physical activity, diabetes self-management, sleep, and stress management. Communication between participants and health coaches was facilitated through secure in-app messaging and scheduled virtual interactions.

#### 2.10.2 Health coach engagement and communication

Behavioral support was delivered by trained health coaches experienced in diabetes lifestyle management. Coaching interactions were conducted through welcome calls, scheduled video consultations, audio follow-up calls, and asynchronous in-app messaging. During these interactions, coaches reviewed participant-generated data, provided individualized feedback, established behavioral goals, addressed barriers to adherence, and reinforced recommended lifestyle practices. Coaching content focused on dietary behavior, physical activity, self-monitoring practices, sleep hygiene, stress management, and long-term behavior change strategies.

#### 2.10.3 Adaptive coaching framework

Intervention delivery followed a stepwise behavioral modification framework. Lifestyle recommendations were introduced progressively according to participant readiness, baseline clinical profile, and behavioral capacity. Adherence was assessed at predefined audit points using engagement and monitoring data captured through the digital platform. Participants demonstrating suboptimal adherence or limited progress received intensified coaching support, including increased communication frequency, additional follow-up interactions, and recalibration of behavioral goals. This adaptive escalation strategy ensured that support intensity remained aligned with individual participant needs throughout the intervention period.

#### 2.10.4 Participant monitoring and engagement tracking

Participant engagement was monitored through both active self-reporting and passive digital data capture. Active monitoring included dietary image submissions, self-monitored blood glucose recordings, body-weight measurements, waist-circumference logging, and participation in coaching sessions. Passive monitoring was achieved through wearable-device synchronization, enabling automated collection of physical activity metrics including daily step count, distance traveled, and active energy expenditure. These data were continuously reviewed through a coach-facing dashboard to support participant specific feedback and adherence monitoring.

### 2.11 Quantitative framework for intervention adherence

Participant compliance was evaluated using a composite, engagement-based adherence framework designed to quantify sustained participation across the multidimensional components of the 90-day intervention. Each domain was assigned a predefined weightage according to its expected participation frequency and clinical relevance, resulting in a maximum monthly adherence score of 100 points. The adherence framework incorporated six domains scored as follows:

- Coaching Engagement (20 points total):
  - Video consultations and welcome calls (10 points; three scheduled sessions per month).
  - Audio follow-up calls (10 points; thrice weekly).
- Glycemic Self-Monitoring (20 points total):
  - Self-monitored blood glucose (SMBG) logging (20 points; minimum of six readings per week).
- Anthropometric Self-Monitoring (18 points total):
  - Weight logging (10 points; thrice weekly).
  - Waist circumference logging (8 points; twice weekly).
- Dietary Logging (12 points total):
  - Food-image submissions (12 points; four submissions per week).
- Structured Exercise Participation (15 points total):
  - Minimum of three strength-based or prerecorded exercise sessions per week (15 points).
- Wearable-Based Physical Activity Tracking (15 points total):
  - Continuous tracking of daily step count and active energy expenditure (15 points).

Collectively, these measures reflected the key behavioral targets of the intervention and enabled comprehensive assessment of participant engagement throughout the study period. Adherence data were obtained from both participant-reported entries and automatically synchronized digital records. Participants received the maximum score allocated to each component when predefined engagement requirements were met, whereas scores were proportionally reduced when engagement fell below the prescribed thresholds. Monthly adherence scores were subsequently aggregated across the intervention period to derive an overall adherence measure for each participant.

For subgroup analyses, participants were categorized according to their overall adherence level. High adherence was defined as the attainment of 35% of the maximum possible adherence score, whereas low adherence was defined as an attainment of <35%. This threshold was established *a priori* based on the study’s structured adherence framework, which graded participation across four tiers: no, minimum, moderate, and maximum adherence.

The 35% threshold was derived from the domain-specific minimum engagement requirements defined within the adherence framework. Each of the six domains specifies a minimum participation threshold necessary to receive partial credit (ranging from 25% for glycemic self-monitoring, anthropometric self-monitoring, dietary logging, structured exercise, and wearable-based activity tracking, to 40% for coaching engagement, reflecting the greater clinical emphasis placed on consistent coach interaction). Averaged across all six domains, this yields a mean minimum engagement threshold of 27.5%. The high-adherence cutoff of 35% was therefore set intentionally above this average, ensuring that participants classified as high-adherence had exceeded not merely the baseline minimum for a single domain, but a meaningfully higher composite engagement level spanning multiple intervention components.

### 2.12 Pre- and post-intervention assessment

At enrollment, baseline demographic and clinical characteristics, including age, sex, duration of diabetes, comorbidities, antihyperglycemic medication history, and associated cardiometabolic profiles, were documented through medical record review and clinical assessment. Vital signs, including systolic and diastolic blood pressure, were recorded using standardized clinical procedures. Anthropometric assessments included height, body weight, BMI, and waist circumference.

Baseline biochemical assessments were performed on fasting venous blood samples collected following a minimum 12-hour overnight fast. Glycemic parameters included HbA1c, FBS, fasting insulin, and C-peptide. Lipid parameters comprised total cholesterol, high-density lipoprotein cholesterol (HDL-C), low-density lipoprotein cholesterol (LDL-C), and triglycerides. Liver function markers included alanine aminotransferase (ALT), aspartate aminotransferase (AST), and alkaline phosphatase (ALP), while renal function was assessed using estimated glomerular filtration rate (eGFR), blood urea nitrogen (BUN), and serum creatinine. HOMA-IR and HOMA-B were subsequently calculated using validated equations based on fasting glucose and insulin concentrations.

Psychological well-being and diabetes-related emotional burden were assessed at baseline using the validated 17-item Diabetes Distress Scale (DDS-17), a widely used instrument for evaluating diabetes-specific emotional distress among individuals with diabetes (22). The DDS comprises four domains: Emotional Distress (ED; 5 items), Physician Distress (PD; 4 items), Regimen Distress (RD; 5 items), and Interpersonal Distress (ID; 3 items). ED reflects feelings of being overwhelmed by the demands of diabetes, PD evaluates concerns related to healthcare provider interactions and support, RD assesses difficulties adhering to diabetes self-management recommendations, and ID captures perceived lack of support from family and social networks. Participants rated each item on a 6-point Likert scale ranging from 1 (“not a problem”) to 6 (“a very serious problem”). Total DDS-17 and domain-specific scores were calculated as mean item scores, with higher scores indicating greater diabetes-related emotional burden. Consistent with established scoring guidelines, mean scores <2.0 indicated little or no distress, scores between 2.0 and 2.9 indicated moderate distress, and scores ≥3.0 indicated high distress. A mean score ≥2.0 was considered indicative of clinically meaningful diabetes distress (22, 23).

Following completion of the 90-day intervention, anthropometric, biochemical, and psychometric assessments were repeated using the same standardized procedures and measurement protocols employed at baseline. Post-intervention assessments included glycemic parameters (HbA1c, and FBS), HOMA indices (HOMA-IR and HOMA-B), anthropometric measures (body weight, BMI, and waist circumference), and psychological outcomes assessed using the DDS-17. Changes between baseline and follow-up measurements were subsequently evaluated to determine the impact of the intervention on metabolic, anthropometric, and psychological outcomes.

To ensure data quality and minimize measurement variability, all assessments were conducted using standardized operating procedures and calibrated instruments throughout the study period.

### 2.13 Sample size calculation

The sample size was calculated *a priori* based on the primary endpoint: absolute change in HbA1c from baseline to 90 days between the Intervention and Control arms. Assuming a clinically meaningful between-group difference of 0.5% (pooled SD = 1.15%), with α = 0.05 (two-sided) and 80% power, approximately 82 evaluable participants per arm were required using the two-sample t-test formula:


$$n=2\times {\left({\text{Z}}_{1}-{\text{α}}_{2}+{\text{Z}}_{1}-\text{β}\right)}^{2}\times {\text{σ}}^{2}/{\text{Δ}}^{2}$$

To account for an anticipated 10% attrition over the 90-day intervention period, the enrollment target was inflated accordingly:


$${\text{N}}_{\text{(adjusted)}}=\text{n}/\left(1-\text{d}\right)$$

The total planned sample size for the trial is therefore N = 90 participants (Intervention = 90; Control = 90).

### 2.14 Statistical analysis

All statistical analyses were performed using R software (version 4.4.2). A two-sided *p*-value <0.05 was considered statistically significant. Comparative efficacy analyses were conducted using a per-protocol approach, restricting the analytical sample to participants who completed the 90-day intervention and follow-up assessments without major protocol deviations. Continuous variables are reported as mean ± standard deviation (SD) and categorical variables as percentages. The analytical population comprised all participants completing both baseline and follow-up assessments.

Distributional normality of continuous variables was assessed using the Shapiro-Wilk test. Baseline comparability between groups was assessed using Welch’s two-sample t-test or the Mann-Whitney U test for continuous variables, depending on normality, while categorical variables such as gender, age and medication distribution were compared using the chi-square test with Yates’ continuity correction or Fisher’s exact test when expected cell counts were <5.

Within-group changes from baseline to follow-up were evaluated using paired t-tests for normally distributed change scores and Wilcoxon signed-rank tests for non-normally distributed variables. Given the observed baseline differences between groups in sex distribution, HbA1c, DDS scores, and HDL cholesterol (Section 3.1), ANCOVA was deliberately employed for all primary and secondary outcome analyses to statistically adjust for baseline values, thereby minimizing the influence of these baseline imbalances on between-group outcome comparisons. The primary efficacy analysis assessing between-group differences in HbA1c reduction was performed using analysis of covariance (ANCOVA), with follow-up HbA1c as the dependent variable, treatment group as the fixed factor, and baseline HbA1c as a covariate. The same ANCOVA framework was applied to all primary and secondary outcomes, including FBS, BMI, body weight, waist circumference, HOMA-IR, HOMA-B, and DDS total and domain scores, with respective baseline values incorporated as covariates. HOMA-based analyses were restricted to participants not receiving exogenous insulin. The magnitude of between-group differences was quantified using Cohen’s d. Notably, no changes to pharmacological therapy were documented during the intervention period in either study group, medication use was considered a fixed baseline characteristic and was not included as a time-varying covariate in the analyses.

Adherence-stratified subgroup analyses were conducted within the intervention cohort by categorizing participants into high-adherence and low-adherence subgroups. Between-group differences in glycemic and anthropometric outcomes were assessed using independent t-tests or Mann-Whitney U tests, as appropriate. A continuous dose-response analysis was additionally performed using Pearson and Spearman correlations and simple linear regression to quantify the relationship between adherence score and HbA1c reduction. These adherence-based analyses were considered exploratory.

## 3 Results

**Figure 2:**
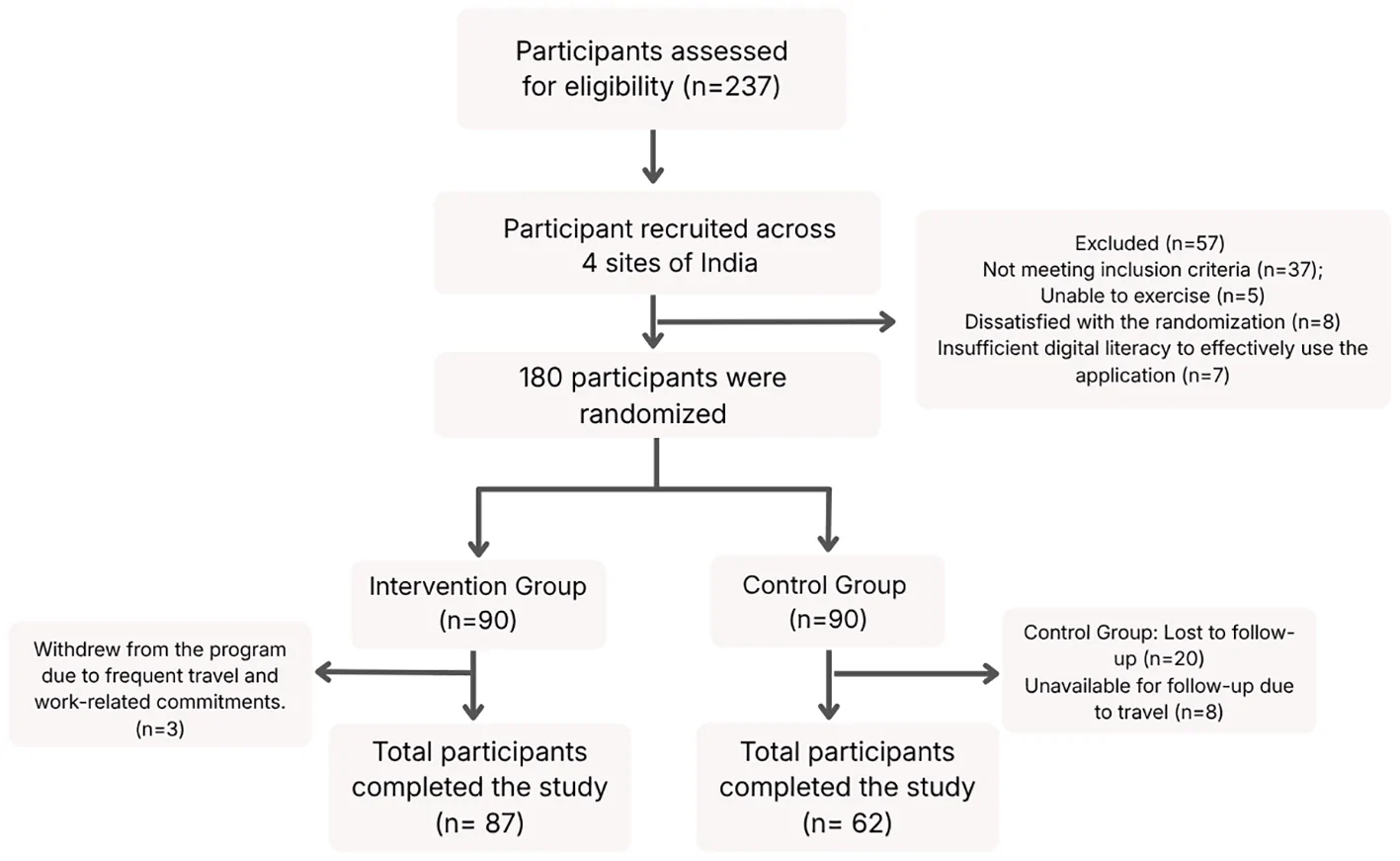
CONSORT flow diagram of participant enrollment, randomization, follow-up, and analysis.

**Figure 2** reports flow diagram of participant screening, allocation, follow-up, and analysis. Of the 237 individuals screened, 57 were excluded based on eligibility criteria, resulting in 180 eligible participants. These participants (n=180) were allocated into the intervention group (n = 90), who received a structured, digitally guided lifestyle program in addition to SOC, and the participants in the control group (n = 90) continued with SOC alone. During the study period, 3 participants from the intervention group and 28 from the control group were lost to follow-up, resulting in 87 (58.39%) and 62 (41.61%) participants completing the study, respectively. In total, 149 participants were included in the final efficacy analyses.

### 3.1 Baseline characteristics of the study cohorts

Overall, the baseline clinical and demographic profiles of the intervention (n=87) and control (n=62) groups were highly consistent (**Table 1**), showing no statistically significant differences in age, duration of diabetes, blood pressure, anthropometric measures, fasting glycemic indices, insulin resistance, β-cell function, or the majority of lipid, hepatic, and renal parameters.

**Table 1:** Baseline characteristics of participants in the intervention and control groups.

Characteristic	Intervention	Control	*p*-value
Demographic and disease characteristics (intervention, n=87; control, n=62)			
Overall Age (years; mean ± SD)	51.5 ± 12.6	55.2 ± 12.2	0.076^a^
Age groups			
20–35 years	9 (10.34%)	6 (9.6%)	1.000^c^
35–50 years	34 (39.1%)	13 (20.96%)	0.03^c^
>50 years	44 (50.57%)	43 (69.35%)	0.034^c^
Male, n (%)	54 (62.1%)	27 (43.5%)	0.038^c^
Duration of diabetes (years; mean ± SD)	10.0 ± 8.0	9.9 ± 7.7	0.946^b^
Glycemic burden (intervention, n=87; control, n=62; mean ± SD)			
HbA1c (%)	9.25 ± 1.82	8.63 ± 1.48	0.036^b^
FBS (mg/dL)	155.92 ± 57.39	148.06 ± 56.83	0.128^b^
Fasting insulin (mIU/L)	12.75 ± 9.79	12.97 ± 8.40	0.566^b^
C-peptide (ng/mL)	2.20 ± 1.15	2.24 ± 0.98	0.567^b^
HOMA indices (intervention, n=66; control, n=49; mean ± SD)			
HOMA-B	50.99 ± 40.77	55.10 ± 82.17	0.2533 ^b^
HOMA-IR	4.64 ± 4.12	4.57 ± 4.25	0.8035 ^b^
Anthropometric status (intervention, n=87; control, n=62; mean ± SD)			
Weight (kg)	74.63 ± 13.18	73.06 ± 11.37	0.783 ^b^
Overall BMI (kg/m^2^)	27.50 ± 4.32	28.36 ± 4.24	0.227^a^
Waist circumference (cm)	96.38 ± 10.95	98.91 ± 10.85	0.165 ^a^
Psychological burden (intervention, n=87; control, n=62; mean ± SD)			
DDS score	2.35 ± 1.15	1.70 ± 0.89	<0.001^b^
Physician Distress (PD)	2.28 ± 1.20	1.77 ± 1.00	0.011^b^
Emotional Distress (ED)	2.28 ± 1.29	1.61 ± 0.87	0.001^b^
Regimen Distress (RD)	2.45 ± 1.27	1.77 ± 0.87	<0.001^b^
Interpersonal Distress (ID)	2.39 ± 1.39	1.70 ± 0.92	0.001^b^
Hemodynamic profile (intervention, n=87; control, n=62; mean ± SD)			
SBP (mmHg)	129.49 ± 11.17	129.98 ± 11.42	0.727^b^
DBP (mmHg)	81.02 ± 7.60	81.00 ± 13.15	0.715^b^
Lipid profile (intervention, n=87; control, n=62; mean ± SD)			
Triglycerides (mg/dL)	167.02 ± 95.76	174.54 ± 85.41	0.3604^b^
HDL-C (mg/dL)	54.45 ± 26.67	41.99 ± 8.72	0.0089^b^
LDL-C (mg/dL)	92.69 ± 38.17	95.10 ± 34.53	0.6881^b^
Total cholesterol (mg/dL)	167.9± 44.57	173.07 ± 42.90	0.4776^b^
Hepatic functional profile (intervention, n=87; control, n=62; mean ± SD)			
ALT (U/L)	31.24 ± 24.18	25.69 ± 18.18	0.093^b^
AST (U/L)	27.38 ± 16.96	24.11 ± 11.74	0.131^b^
ALP (U/L)	82.84 ± 24.33	84.44 ± 30.06	0.998^b^
Renal functional profile (intervention, n=87; control, n=62; mean ± SD)			
eGFR (mL/min/1.73m^2^)	102.19 ± 15.51	96.24 ± 20.08	0.0843^b^
BUN (mg/dL)	11.24 ± 3.88	11.13 ± 4.66	0.6263^b^
Creatinine (mg/dL)	0.79 ± 0.19	0.79 ± 0.29	0.2354^b^
Antihyperglycemic medication exposure, n (%)			
Biguanides	79 (90.8%)	51 (82.3%)	0.1962^c^
Sulfonylureas	53 (60.9%)	44 (71.0%)	0.2739^c^
DPP-4 inhibitors	52 (59.8%)	37 (59.7%)	1^c^
SGLT2 inhibitors	26 (29.9%)	27 (43.5%)	0.1227^c^
Insulin	21 (23.0%)	13 (21.0%)	0.9262^c^
Alpha-glucosidase inhibitors	17 (19.5%)	23 (37.1%)	0.0281^c^
TZDs	14 (16.1%)	7 (11.3%)	0.5542^c^
GLP-1 receptor agonists	1 (1.1%)	2 (3.2%)	0.5707^d^
Meglitinides	1 (1.1%)	2 (3.2%)	0.5707^d^
Dual PPAR agonists	1 (1.1%)	1 (1.6%)	1^d^

Data are presented as mean ± standard deviation (SD) for continuous variables and/or n (%) for categorical variables.

^a^ *p* values evaluated by Welch’s two-sample t-test.

^b^ *p* values evaluated by Mann–Whitney U test.

^c^ *p* values evaluated by chi-square test.

^d^ *p* value evaluated by Fisher’s exact test.

HOMA-B and HOMA-IR were calculated only among participants not receiving exogenous insulin therapy (intervention, n = 66; control, n = 49). *p*-values represent between-group comparisons at baseline. HbA1c, glycated hemoglobin; FBS, fasting blood sugar; HOMA-B, homeostatic model assessment of β-cell function; HOMA-IR, homeostatic model assessment of insulin resistance; BMI, body mass index; DDS, Diabetes Distress Scale; PD, physician distress; ED, emotional distress; RD, regimen distress; ID, interpersonal distress; SBP, systolic blood pressure; DBP, diastolic blood pressure; HDL-C, high-density lipoprotein cholesterol; LDL-C, low-density lipoprotein cholesterol; ALT, alanine aminotransferase; AST, aspartate aminotransferase; ALP, alkaline phosphatase; eGFR, estimated glomerular filtration rate; BUN, blood urea nitrogen.

Nevertheless, a few baseline differences were observed. The intervention group included a greater proportion of male participants and had slightly higher mean height values than the control group. In addition, baseline HbA1c levels were modestly higher in the intervention group, indicating a greater degree of chronic glycemic exposure at enrollment. Despite this difference, fasting glucose, fasting insulin, C-peptide, HOMA-IR, and HOMA-B values were comparable between groups, suggesting similar underlying insulin resistance, endogenous insulin secretory capacity, and β-cell functional status at study baseline. Assessment of β-cell function was restricted to participants not receiving exogenous insulin therapy (intervention group: n=66; control group: n=49). Participants in the intervention group exhibited higher baseline DDS scores and HDL-C concentrations compared with those in the control group.

Both cohorts were receiving active pharmacological management for diabetes at baseline, with broadly comparable exposure to antihyperglycemic therapies. In both the intervention and control groups, biguanides, sulfonylureas, and dipeptidyl peptidase-4 (DPP-4) inhibitors constituted the primary, most frequently utilized therapeutic classes. Moderate utilization was noted across both arms for sodium-glucose cotransporter 2 (SGLT2) inhibitors, insulin, alpha-glucosidase inhibitors, and thiazolidinediones (TZDs). Conversely, prescription rates for glucagon-like peptide-1 (GLP-1) receptor agonists, meglitinides, and dual peroxisome proliferator-activated receptors (PPAR) agonists remained relatively limited in both cohorts. Although modest variations were observed in the utilization of specific drug classes, the similarity in diabetes duration, fasting glycemic measures, insulin resistance, β-cell function, and overall treatment intensity suggests that the two groups represented clinically comparable populations receiving routine diabetes care.

### 3.2 Primary outcome analysis: comparative analysis of changes between intervention and control groups

**Figure 3:**
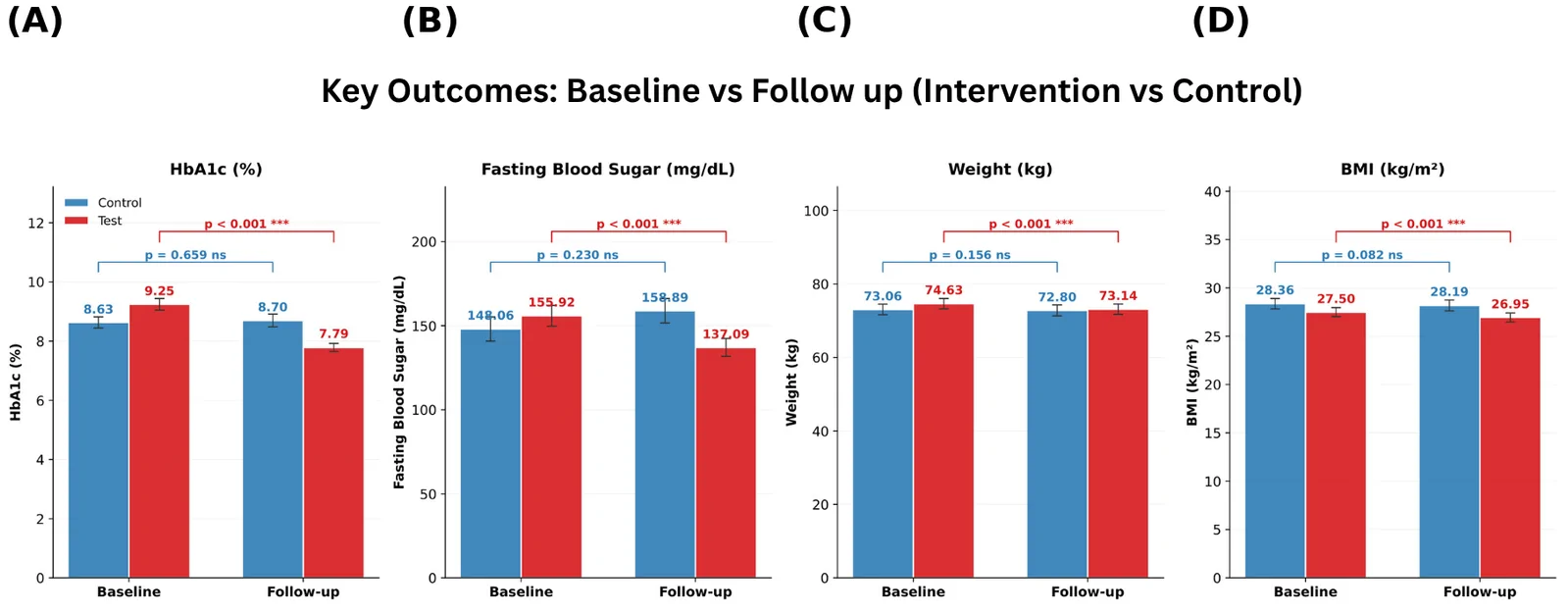
Changes in primary outcomes following the 90-day lifestyle intervention. Comparison of changes in primary study outcomes between the intervention and control groups over 90 days. Data are presented as mean changes from baseline for glycemic parameters **(A)** HbA1c and **(B)** FBS and anthropometric measures **(C)** Body weight and **(D)** BMI. Error bars represent standard deviations. Statistical significance was assessed between-group comparisons.

As presented in **Table 2** and **Figure 3**, the primary efficacy analyses demonstrated robust improvements in both glycemic and anthropometric outcomes following the 90-day lifestyle intervention. Within the intervention cohort, participants achieved highly significant reductions in both HbA1c and FBS over the study period (both p < 0.001). Conversely, the control group exhibited no meaningful improvement in glycemic control; their average HbA1c remained statistically unchanged (p = 0.65), while FBS showed a slight, non-significant upward trend (p = 0.23). Reflecting these opposing trajectories, between-group comparisons confirmed that the intervention group achieved significantly greater reductions in both HbA1c (p < 0.001) and FBS (p = 0.0014) relative to those receiving standard care alone.

**Table 2:** Changes in glycemic and anthropometric outcomes and between-group comparisons following the 90-day lifestyle intervention.

Variables	Change from baseline (intervention group; mean ± SD)			Change from baseline (control group; mean ± SD)			Cohen’s d	Between-group *p*-value
	Follow-up	Mean change	*P*-value	Follow-up	Mean change	*P*-value		
HbA1c (%)	7.79 ± 1.27	+1.46 ± 1.52	<0.00 1^b^	8.70 ± 1.71	-0.07 ± 1.22	0.65^b^	1.09	<0.001^c^
FBS (mg/dL)	137.09 ± 49.64	+18.83 ± 60.46	<0.00 1^b^	158.89 ± 57.44	-10.84 ± 55.20	0.23^b^	0.51	0.0014^c^
BMI (kg/m^2^)	26.95 ± 4.32	+0.55 ± 0.85	<0.00 1^b^	28.19 ± 4.42	+0.18 ± 0.78	0.08^a^	0.46	0.0026^c^
Weight (kg)	73.14 ± 13.11	+1.49 ± 2.22	<0.00 1^b^	72.80 ± 11.76	+0.26 ± 2.57	0.15^b^	0.52	<0.001^c^

Data are presented as mean ± standard deviation.

^a^ *p* values evaluated by Paired t test.

^b^ *p* values evaluated by Wilcoxon signed-rank.

^c^ *p* values evaluated by ANCOVA.

Cohen’s d values indicate the magnitude of the between-group’s intervention effect.

HbA1c, glycated hemoglobin; FBS, fasting blood sugar; and BMI, body mass index.

Anthropometric markers followed a similar positive trend. Within-group analyses revealed that the intervention group experienced significant reductions in both body mass index (BMI) and total body weight by Day 90 (both p < 0.001). In contrast, changes within the control group were marginal and did not reach statistical significance for either BMI (p = 0.08) or body weight (p = 0.15). Consequently, between-group analyses confirmed that the lifestyle intervention was significantly more effective at driving weight loss and reducing BMI than standard care (p < 0.001 and p = 0.0026, respectively).

### 3.3 Secondary outcome analysis: comparing changes between intervention and control groups

As presented in **Table 3**, secondary outcome analyses evaluated changes from baseline to follow-up in anthropometric, homeostatic, and psychosocial outcomes following the 90-day lifestyle intervention. Compared with the control group, participants in the intervention group exhibited significantly greater improvements across several secondary outcome measures.

**Table 3:** Changes in waist circumference, markers of insulin homeostasis, and diabetes distress outcomes following the 90-day lifestyle intervention.

Parameter	Change from baseline (intervention group)			Change from baseline (control group)			Cohen’s d	Between-group *p*-value
	Follow-up	Mean change	*p*-value	Follow-up	Mean change	*p*-value		
Waist circumference (cm)	94.52 ± 10.45	+1.86 ± 2.53	<0.001^a^	99.00 ± 10.84	-0.09 ± 3.93	0.7364^a^	0.61	0.0001^c^
HOMA-IR	4.27±6.34	+0.36 ± 6.63	0.0038 ^a^	4.83 ± 5.62	-0.27±4.58	0.9620 ^a^	0.108	0.0481 ^c^
HOMA-B	87.27 ± 14 1.10	+36.28±134.32	0.001 9 ^a^	63.81 ± 57.04	+8.72± 79.1	0.8906 ^a^	0.241	0.0556 ^c^
Overall DDS score	1.78 ± 0.81	+0.57 ± 0.77	<0.00 1 ^a^	1.97 ± 0.77	-0.27 ± 0.71	<0.001 ^a^	1.13	<0.001 ^c^
PD	1.80 ± 0.8 9	+0.48 ± 0.8 7	<0.00 1 ^b^	1.97± 0.79	-0.19 ± 0. 84	0.0548 ^b^	0.81	<0.001 ^c^
ED	1.74 ± 0.8 7	+0.54 ± 0.9 4	<0.00 1 ^b^	1.88± 0.78	-0.27 ± 0. 69	0.003 ^b^	0.99	<0.001 ^c^
RD	1.83± 0.91	+0.62 ± 0.9 2	<0.00 1 ^b^	2.01± 0.81	-0.24 ± 0. 82	0.0128 ^b^	1.02	<0.001 ^c^
ID	1.75 ± 0.9 1	+0.63 ± 1.1 4	<0.00 1 ^b^	2.07± 0.94	-0.38 ± 0. 82	0.0003 ^b^	1.05	<0.001 ^c^

Data are presented as mean ± standard deviation.

^a^ *p* values evaluated by Wilcoxon signed-rank.

^b^ *p* values evaluated by Paired t test.

^c^ *p* values evaluated by ANCOVA.

Cohen’s d values indicate the magnitude of the between-group’s intervention effect.

HOMA-IR, Homeostatic Model Assessment of Insulin Resistance; HOMA-B, Homeostatic Model Assessment of β-cell Function; DDS, Diabetes Distress Scale; PD, Physician Distress; ED, Emotional Distress; RD, Regimen Distress; ID, Interpersonal Distress.

Waist circumference decreased significantly in the intervention group (p < 0.001), whereas the control group showed no significant change over the 90-day period (p = 0.7364). Consequently, the reduction in waist circumference was significantly greater in the intervention group compared with the control group (p < 0.001).

Markers of insulin homeostasis also demonstrated favorable changes. HOMA-IR decreased significantly in the intervention group (p = 0.0038), whereas the control group experienced no statistically significant change (p = 0.9620). This difference between the two groups was statistically significant (p = 0.0481). Similarly, HOMA-B increased significantly within the intervention group (p = 0.0019), whereas the control group demonstrated a small, non-significant increase (p = 0.8906). Although the magnitude of within-group improvement was greater in the intervention arm, the final between-group difference did not reach statistical significance (p = 0.0556).

Significant improvements were also observed in diabetes-related distress. In the intervention group, the overall DDS score decreased significantly (p < 0.001), whereas the control group experienced a significant increase in distress (p < 0.001). This resulted in a highly significant between-group difference in favor of the intervention (p < 0.001).

This positive pattern was consistent across all individual DDS domains. Within the intervention group, statistically significant reductions were achieved across all subscales: PD, ED, RD, and ID (all p < 0.001). In contrast, the control group exhibited marked increases in distress from baseline to post-intervention, with statistically significant worsening observed across all subscales: PD (p = 0.0548), ED (p = 0.003), RD (p = 0.0128), and ID (p = 0.0003). Overall, between-group analyses demonstrated significantly greater improvements in overall and domain-specific diabetes-related distress outcomes among participants receiving the digital lifestyle intervention compared to those receiving standard care alone.

### 3.4 Adherence-based subgroup analysis on glycemic and anthropometric outcomes

**Figure 4:**
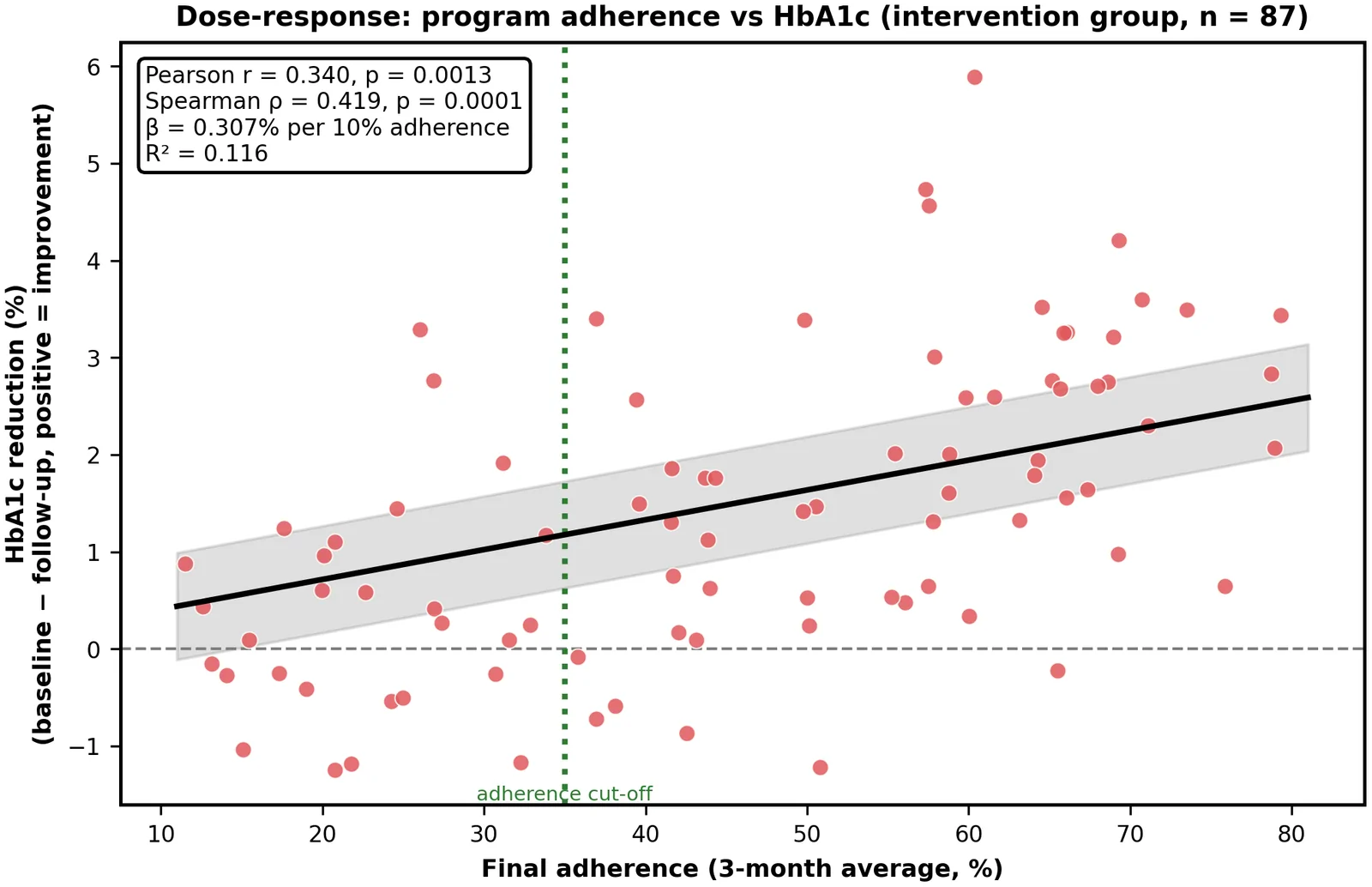
Subgroup analysis: effect of program adherence on HbA1c reduction among participants in the intervention group. High adherence, ≥35% of the maximum possible adherence score; low adherence, <35% of the maximum possible adherence score. The ≥35% threshold was established *a priori* to represent the minimum level of engagement considered necessary for meaningful exposure to multiple intervention components.

To evaluate the relationship between engagement levels and clinical response, the intervention cohort was stratified into high-adherence (n=62) and low-adherence (n=25) subgroups. Baseline demographic and anthropometric characteristics remained broadly comparable between these adherence strata, facilitating a direct evaluation of adherence-associated variations in intervention response (**Table 4**; **Figure 4**).

**Table 4:** Glycemic and anthropometric outcomes among high- and low-adherence participants receiving the digital precision care intervention.

Parameter	Participants with high adherence (n=62)				Participants with low adherence (n=25)				Between-group *p*-value
	Baseline	Follow-up	Mean change	*p*-value	Baseline	Follow-up	Mean change	*p*-value	
HbA1c (%)	9.3 ± 1.9	7.6 ± 1.2	+1.7 ± 1.5	<0.05^a^	9.0±1.6	8.2±1.4	+0.8±1.3	<0.05 ^a^	<0.05^b^
FBS (mg/dL)	155.5±58.5	130.3±46.5	+25.2±60.0	<0.05 ^a^	156.9±55.7	153.8±54.2	+3.1±59.9	>0.05 ^a^	<0.05 ^b^
BMI (kg/m^2^)	27.4± 4	26.9±4.1	+0.6±+0.9	<0.05 ^a^	27.6±5.0	27.1±4.8	+0.5±+0.8	<0.05 ^a^	>0.05 ^b^
Weigh t (kg)	74.7± 12.7	73.1± 12.8	+1.6± 2.3	<0.05 ^a^	74.7± 14.5	73.3± 14.0	+1.4± 2.1	<0.05 ^a^	<0.05 ^b^

^a^ *p* values evaluated by Wilcoxon signed-rank.

^b^ *p* values evaluated by Mann-Whitney U.

HbA1c, glycated hemoglobin; FBS, fasting blood sugar; BMI, body mass index.

A progressive improvement in glycemic outcomes was observed with higher adherence to the lifestyle program. Participants with high adherence achieved a substantial and statistically significant reduction in HbA1c (p < 0.05). In contrast, those with low adherence demonstrated a smaller, though still significant, reduction (p < 0.05). This adherence-associated trend was similarly reflected in FBS outcomes. Participants with high adherence showed a significant reduction in FBS (p < 0.05), whereas those with low adherence demonstrated minimal, non-significant changes (p > 0.05).

In contrast to the glycemic trends, favorable improvements in anthropometric parameters were observed across both adherence subgroups from baseline to post-intervention. Participants with high adherence achieved a significant reduction in BMI (p < 0.05), while the low-adherence group experienced a statistically similar decrease (p < 0.05). Body weight also decreased significantly in both strata, with the high-adherence subgroup achieving a significantly greater reduction than the low-adherence group.

Ultimately, between-group comparisons revealed that the high-adherence subgroup achieved significantly greater improvements in HbA1c (p<0.05), FBS (p<0.05), and overall body weight (p<0.05) compared to the low-adherence subgroup. In contrast, no statistically significant between-group difference was observed for changes in BMI (p>0.05). This clear adherence-response relationship links higher digital engagement directly to superior glycemic and anthropometric outcomes.

## 4 Discussion

This trial addresses a notable gap in the Indian T2D literature, where evidence on structured, digitally delivered, multicomponent lifestyle interventions, as opposed to single-component or purely app-based tools, remains limited, despite India’s high T2D burden and well-documented shortfall in structured lifestyle counseling within routine care. To evaluate this strategy, our RCT demonstrated that, compared with SOC, an integrated, multicomponent lifestyle program, significantly optimized glycemic, anthropometric, and metabolic profiles in individuals with T2D. The intervention cohort achieved significantly greater reductions in HbA1c, FBS, body weight, waist circumference, HOMA-IR, and HOMA-B than the control group, which exhibited non-significant changes across these parameters. Furthermore, subgroup analyses demonstrated a clear engagement-response relationship; participants with high adherence achieved progressively superior reductions in glycemic and anthropometric parameters, while those with lower program adherence still experienced favorable, meaningful improvements. Collectively, these findings indicate that a multidimensional, digitally enabled lifestyle intervention can produce substantial metabolic and anthropometric optimization when delivered alongside routine pharmacological diabetes care.

The specific glycemic trajectories and timing observed in the present study are broadly consistent with established trends in structured care literature. Prior intervention data demonstrate that multimodal digital health platforms and community-based healthcare initiatives yield significant, sustained HbA1c reductions among participants who maintain consistent program engagement compared to standard care (24, 25). Furthermore, evidence from the integrated personalized diabetes management (iPDM) framework indicates that the velocity of this therapeutic HbA1c reduction is greatest during the first three months of intervention and is subsequently maintained during longer-term follow-up (26). In addition to glycemic improvements, significant reductions in anthropometric parameters were observed in the intervention group. These findings align with previous studies demonstrating that structured lifestyle interventions can improve measures of adiposity and central obesity. Although some interventions have reported limited changes in body weight, significant reductions in waist circumference have been observed among participants with higher adherence to lifestyle recommendations (27). In our cohort, this dual optimization of both glycemic and anthropometric measures underscores the holistic metabolic impact of the digital platform.

This comprehensive clinical efficacy is driven by the structural frameworks of the digital tools themselves. Consistent with recent data, randomized and observational studies indicate that multi-component digital interventions incorporating app-based lifestyle coaching, tailored actionable recommendations, structured education, physical activity guidance, and continuous behavioral support consistently drive marked improvements in glycemic outcomes and are strongly associated with improved self-management behaviors compared with usual care (28, 29). These findings are further reinforced by clinical evaluations demonstrating that integrating technical monitoring features, such as remote glucose monitoring and medication tracking, with continuous motivational support substantially enhances patient behavioral accountability and overall metabolic profiles (30).

Collectively, these distinct lines of clinical evidence underscore a critical insight: the therapeutic effectiveness of digital health programs relies heavily on the synergistic integration of multiple, evidence-based behavioral strategies within an integrated care framework, rather than the deployment of isolated interventions. By simultaneously addressing dietary modifications, physical activity, self-monitoring, and behavioral coaching, these multidimensional digital tools offer an effective, scalable adjunct to conventional pharmacological diabetes care. This trial was intentionally designed around the principle that sustainable glycemic and behavioral improvement in T2D is rarely achieved through any single intervention lever, but through an integrated combination of nutrition, physical activity, structured eating patterns, and continuous behavioral coaching. This design philosophy, treating the participant holistically rather than isolating and testing single variables, reflects how real-world, comprehensive diabetes care is intended to function, and is consistent with pragmatic trial designs evaluating similarly structured, multicomponent digital health programs. A natural consequence of this integrated approach is that the relative statistical contribution of individual components (dietary modification, physical activity, time-restricted eating, or coaching engagement) to the observed outcomes cannot be isolated within this trial design; disentangling these effects would require a factorial or dismantling trial, which represents a valuable direction for future research building on this integrated model.

Reflecting this multi-component approach, several factors may explain the superior outcomes observed in our intervention cohort compared with SOC, driven by an integrated and adaptive framework addressing both physiological and behavioral determinants of diabetes management. For instance, structured dietary guidance promoted healthier eating patterns, while structured physical activity guidance, reinforced through empathetic coaching interactions helped participants overcome daily barriers and maintain long-term engagement. Furthermore, the incorporation of a human-in-the-loop coaching model established continuous communication between participants and the care team. This enabled the timely resolution of challenges, provision of tailored guidance, and adaptive adjustment of recommendations in response to individual circumstances, thereby strengthening participant accountability. Concurrently, continuous self-monitoring and real-time feedback enabled the iterative refinement of care plans throughout the 90-day intervention period, ensuring alignment with patient progress and reinforcing the behavioral consistency necessary to maximize clinical efficacy.

A notable finding of the present study was the clear adherence-response relationship within the intervention cohort. Exploratory subgroup analyses demonstrated that the participants with high adherence achieved substantially greater reductions in HbA1c and FBS than those with lower adherence. These findings indicate that the magnitude of clinical improvement is closely linked to sustained engagement with the intervention components. While traditional lifestyle modification trials report that participant compliance fluctuates significantly across behavioral domains, showing good adherence to balanced diets and physical activity but less consistency toward stress management, regular medical checkups, and medication timelines (31). Our study systematically quantified overall program engagement to evaluate its direct impact on metabolic trends. This granular approach to tracking adherence is particularly relevant in the Indian context, where diabetes self-management is frequently challenged by difficulties in maintaining dietary modifications, sustaining physical activity, limited access to structured diabetes education, and competing personal and occupational responsibilities (32–34). Multi-component digital interventions help directly overcome these localized barriers by substituting fragmented, self-reported habits with continuous monitoring, individualized feedback, and regular coaching interactions.

The observed dietary logging adherence levels are consistent with well-documented underreporting and logging fatigue associated with self-reported dietary tracking methods generally. Coach-reported qualitative observations suggested that actual dietary engagement may have exceeded what was captured through photo submissions alone, though this could not be independently verified. This observation reinforces the rationale for this program’s multi-domain adherence framework, spanning coaching engagement, glycemic and anthropometric self-monitoring, structured exercise, and dietary logging, designed to capture a composite picture of engagement rather than rely on any single, individually imperfect, self-reported metric.

The TRE window was applied as a universal structural component across all participants, while macronutrient composition and food choices within that window followed structured dietary guidance. We view these as complementary rather than conflicting design elements: TRE provided a consistent temporal eating structure, within which structured nutrition guidance was layered, though we acknowledge that the optimal integration of structured eating-window interventions with fully flexible, guideline-based nutrition therapy warrants further investigation.

Beyond scalability, these findings highlight the importance of self-management and active patient participation in diabetes care. Previous research suggests that individuals with T2D often possess adequate knowledge regarding disease management but frequently struggle to translate that knowledge into sustained healthy behaviors (35). This knowledge-behavior gap continues to represent a key barrier in long-term diabetes control. The present intervention addressed this gap by encouraging active participation in goal setting, problem solving, and daily health-related decision-making, thereby strengthening patient ownership of disease management. This increased engagement may have improved awareness of dietary intake, physical activity patterns, and glycemic responses, enabling more informed lifestyle choices. Such changes may have facilitated the adoption and maintenance of healthier behaviors over time (36).

Parallel to these behavioral adaptations, optimizing psychological well-being played a vital complementary role in driving our trial’s clinical success. Given that diabetes-related distress frequently undermines a patient’s capacity for consistent self-care, the meaningful reductions in emotional distress observed within our intervention cohort represent a key therapeutic mechanism that directly enhanced patient self-efficacy. These outcomes strongly align with established literature demonstrating that structured psychological interventions significantly improve both emotional well-being and objective glycemic control in individuals with T2D (37). Specifically, targeted behavioral therapies have been shown to mitigate anxiety and depressive symptoms while concurrently boosting treatment adherence and daily self-management capacity (38, 39). This dual metabolic and emotional efficacy is further substantiated by robust meta-analytic data (40). Although the independent contribution of the CBT component was not separately quantified in this study, the significant reduction in diabetes distress directly complemented the nutritional and physical activity components to optimize overall clinical outcomes.

The findings of this study support the growing role of digitally enabled lifestyle medicine as an adjunct to conventional diabetes care. The integration of behavioral analytics, individualized tracking, real-time feedback, and continuous remote coaching enables scalable delivery of patient centered care beyond traditional clinical settings. Importantly, the combination of digital tools with human-guided coaching may enhance motivation, accountability, and sustained behavior change, which are critical for long-term diabetes management. These results also highlight the importance of active patient participation and self-management in achieving optimal outcomes. By engaging participants in goal setting, problem solving, and daily decision-making, the intervention helped bridge the well-established knowledge-behavior gap in diabetes care. The observed improvements in both metabolic and psychological domains further support the potential of integrated digital interventions as scalable and patient-centered models of care delivery.

Participants in this trial were recruited exclusively from specialized, private-sector diabetes care centers across four Indian cities, rather than from primary care or community health settings. Patients attending such specialty clinics may differ systematically from the broader Indian T2D population, specifically regarding disease duration, access to specialist care, health-seeking behavior, and socioeconomic status, given that consultations and lifestyle counseling at these centers are typically borne out-of-pocket. Consequently, findings from this cohort may not be directly generalizable to patients managed exclusively within primary care or lower-resource public health settings, where baseline engagement with structured diabetes care, and consequently openness to a structured digital lifestyle program, may differ.

Additionally, neither digital literacy nor health literacy was formally assessed at baseline, representing a limitation in characterizing the study population. However, several design features of the intervention were intended to minimize dependence on participants’ baseline digital proficiency. Rather than relying solely on self-directed app navigation, the program incorporated proactive, coach-initiated phone calls as a primary engagement mechanism, and the core digital task required of participants, submitting meal photographs, used a simple, chat-based interface analogous to WhatsApp, a messaging platform with near-ubiquitous adoption among Indian smartphone users regardless of formal digital literacy. Health coaches were further matched to participants by geographic location and language preference, enabling multilingual, culturally contextualized communication intended to enhance trust, comprehension, and behavioral engagement independent of participants’ technical sophistication. While these design elements were intended to lower the effective digital literacy burden, we acknowledge that the absence of formal digital and health literacy assessment limits our ability to quantify their impact on outcomes, and represents an important area for measurement in future iterations of this program.

### 4.1 Strength and limitations

This study’s primary strength lies in its internally validated, randomized controlled design, which minimized residual confounding through robust baseline comparability, allowing for comprehensive multidimensional outcome profiling and an adherence-stratified analysis demonstrating a clear dose-response relationship between program engagement and improvements across both metabolic and psychological domains. No serious adverse events or severe hypoglycemic episodes were observed, and the intervention framework builds on prior real-world evidence (41), supporting its translational relevance for clinical implementation.

Despite these strengths, several limitations should be acknowledged. First, the 90-day duration precludes assessment of long-term durability; extended follow-up is needed to determine sustainability and impact on chronic complications. Second, as a self-management paradigm, efficacy is inherently dependent on patient-driven adherence, though the observed retention and adherence rates support the intervention’s feasibility and acceptability. Third, allocation was operationalized through centrally generated, site-administered cue cards rather than electronic randomization; while concealment was preserved through independent CRC administration, centralized electronic randomization may better streamline future multicenter iterations of this protocol.

Fourth, despite randomization, statistically significant baseline differences were observed between groups in sex distribution, HbA1c, DDS, and HDL cholesterol (**Table 1**). ANCOVA adjustment for each outcome’s baseline value was applied throughout to account for these differences, though residual confounding cannot be fully excluded. Propensity score methods, while a possible alternative, are primarily suited to observational designs; given our sample size and the limited number of imbalanced covariates, ANCOVA adjustment was considered the more appropriate approach for this trial, with propensity-based sensitivity analyses noted as a direction for future, larger-scale studies.

Fifth, SOC was not formally standardized across the four participating centers, consistent with routine Indian diabetes care, where structured lifestyle counseling is discretionary, out-of-pocket, and inconsistently prioritized, and physician contact is typically limited to quarterly visits. This reflects the pragmatic, real-world nature of the comparator and underscores the clinical rationale for structured, continuous lifestyle support, though residual inter-site variability in usual care cannot be excluded.

The observed dietary logging adherence levels are consistent with well-documented underreporting and logging fatigue associated with self-reported dietary tracking methods generally. Coach-reported qualitative observations suggested that actual dietary engagement may have exceeded what was captured through photo submissions alone, though this could not be independently verified. This observation reinforces the rationale for this program’s multi-domain adherence framework, spanning coaching engagement, glycemic and anthropometric self-monitoring, structured exercise, and dietary logging, designed to capture a composite picture of engagement rather than rely on any single, individually imperfect, self-reported metric. Consequently, quantitative energy and macronutrient intake data could not be reported. This represents a significant methodological limitation: in the absence of quantified dietary intake data, the extent to which observed outcomes reflect dietary modification specifically, as opposed to other intervention components, cannot be determined, and findings should be interpreted with appropriate caution regarding the specific mechanisms driving the observed effects.

## 5 Conclusion

This study demonstrates that an integrated, multicomponent lifestyle program, digitally supported lifestyle intervention integrating nutrition, physical activity, psychological support, and continuous human-guided coaching can significantly improve glycemic control and anthropometric outcomes in individuals with T2D. The observed adherence-response relationship highlights the critical role of sustained participant engagement in driving clinical benefit, while the integration of empathetic coaching and real-time feedback underscores the value of combining digital platforms with structured human interaction. Importantly, favorable changes were also observed in measures reflecting endogenous insulin dynamics and diabetes-related emotional burden, indicating broader benefits beyond glycemic and anthropometric outcomes alone. The use of standardized, remotely deliverable protocols, continuous digital monitoring, and structured coaching workflows further supports the potential for broader implementation across diverse healthcare settings with reduced reliance on in-person clinical visits. Collectively, these features indicate that this model represents an effective and adaptable strategy for comprehensive diabetes management.

## Data Availability

The original contributions presented in the study are included in the article/supplementary material. Further inquiries can be directed to the corresponding author.

## References

[1] International Diabetes Federation . Diabetes Atlas (2024). Available online at: https://diabetesatlas.org/data-by-location/global/ (Accessed May 16, 2026).

[2] Chauhan S Khatib MN Ballal S Bansal P Bhopte K Gaidhane AM . The rising burden of diabetes and state-wise variations in India: insights from the Global Burden of Disease Study 1990-2021 and projections to 2031. Front Endocrinol. (2025) 16:1505143. doi: 10.3389/fendo.2025.1505143 40421244 PMC12104079

[3] Finaviya KB Mehta PB Bhut YR . Impact of lifestyle interventions versus pharmacological therapy on glycemic control and metabolic parameters in type 2 diabetes. Int J Curr Pharm Rev Res. (2025) 17:886–90.

[4] Borgharkar SS Das SS . Real-world evidence of glycemic control among patients with type 2 diabetes mellitus in India: the TIGHT study. BMJ Open Diabetes Res Care. (2019) 7:e000654. doi: 10.1136/bmjdrc-2019-000654 31413840 PMC6673766

[5] Avramidis I Apsemidou A Lalia AZ Petridis N Tourtouras E Kalopitas G . Lessons from a diabetes clinic: achieving glycemic goals and clinical use of antidiabetic agents in patients with type 2 diabetes. Clin Diabetes. (2020) 38:248–55. doi: 10.2337/cd19-0090 32699473 PMC7364453

[6] Mendes R Martins S Fernandes L . Adherence to medication, physical activity and diet in older adults with diabetes: its association with cognition, anxiety and depression. J Clin Med Res. (2019) 11:583–92. doi: 10.14740/jocmr3894 31413770 PMC6681861

[7] Davies MJ Aroda VR Collins BS Gabbay RA Green J Maruthur NM . Management of hyperglycemia in type 2 diabetes, 2022: a consensus report by the American Diabetes Association (ADA) and the European Association for the Study of Diabetes (EASD). Diabetes Care. (2022) 45:2753–86. doi: 10.2337/dci22-0034 36148880 PMC10008140

[8] Bajaj S . RSSDI clinical practice recommendations for the management of type 2 diabetes mellitus 2017. Int J Diabetes Dev Ctries. (2018) 38:1–115. doi: 10.1007/s13410-018-0604-7 29527102 PMC5838201

[9] Knowler WC Barrett-Connor E Fowler SE Hamman RF Lachin JM Walker EA . Reduction in the incidence of type 2 diabetes with lifestyle intervention or metformin. N Engl J Med. (2002) 346:393–403. doi: 10.1056/NEJMoa012512 11832527 PMC1370926

[10] Franz MJ Boucher JL Rutten-Ramos S VanWormer JJ . Lifestyle weight-loss intervention outcomes in overweight and obese adults with type 2 diabetes: a systematic review and meta-analysis of randomized clinical trials. J Acad Nutr Diet. (2015) 115:1447–63. doi: 10.1016/j.jand.2015.02.031 25935570

[11] de Hoogh IM Oosterman JE Otten W Krijger AM Berbée-Zadelaar S Pasman WJ . The effect of a lifestyle intervention on type 2 diabetes pathophysiology and remission: the Stevenshof pilot study. Nutrients. (2021) 13:2193. doi: 10.3390/nu13072193 34202194 PMC8308398

[12] Fathima SN . Optimizing diabetes outcomes through evidence-based counseling: a framework for personalized and sustainable self-management. In: Mohammed IA Sharma AVNS Vettriselvan R Airen V Saxena R Ojah AK , editors. Foundations and Futures of Multidisciplinary Research: Cross-Domain Methods and Models. Integrated Publications, New Delhi (2024). p. 78–84. doi: 10.25215/9371832592.10

[13] Ghosh A Banerjee S Dalai CK Chaudhuri S Sarkar K Sarkar D . Medication adherence and environmental barriers to self-care practice among people with diabetes: a cross-sectional study in a lifestyle clinic in eastern India. J Fam Med Prim Care. (2023) 12:325–31. doi: 10.1016/j.jtumed.2023.01.010 36852344 PMC9958071

[14] Kerr D Ahn D Waki K Wang J Breznen B Klonoff DC . Digital interventions for self-management of type 2 diabetes mellitus: systematic literature review and meta-analysis. J Med Internet Res. (2024) 26:e55757. doi: 10.2196/55757 39037772 PMC11301119

[15] Hohberg V Kreppke JN Kohl J Seelig E Zahner L Streckmann F . Effectiveness of a personal health coaching intervention (diabetescoach) in patients with T2D: protocol for an open-label, pragmatic randomised controlled trial. BMJ Open. (2022) 12:e058368. doi: 10.1136/bmjopen-2021-058368 35649615 PMC9161069

[16] Ulambayar B Ghanem AS Tóth Á Nagy AC . Impact of physical activity and dietary habits on mental well-being in patients with diabetes mellitus. Nutrients. (2025) 17:1042. doi: 10.3390/nu17061042 40292462 PMC11944386

[17] Schulz KF Altman DG Moher D CONSORT Group . CONSORT 2010 statement: updated guidelines for reporting parallel group randomised trials. BMJ. (2010) 340:c332. doi: 10.1136/bmj.c332 20332509 PMC2844940

[18] Eysenbach G CONSORT-EHEALTH Group . CONSORT-EHEALTH: improving and standardizing evaluation reports of web-based and mobile health interventions. J Med Internet Res. (2011) 13:e126. doi: 10.2196/jmir.1923 22209829 PMC3278112

[19] Misra A Chowbey P Makkar BM Vikram NK Wasir JS Chadha D . Consensus statement for diagnosis of obesity, abdominal obesity and the metabolic syndrome for Asian Indians and recommendations for physical activity, medical and surgical management. J Assoc Physicians India. (2009) 57:163–70. 19582986

[20] Misra A Vikram NK Ghosh A Ranjan P Gulati S India Obesity Commission Members . Revised definition of obesity in Asian Indians living in India. Diabetes Metab Syndr. (2025) 19:102989. doi: 10.1016/j.dsx.2024.102989 39814628

[21] Food and Agriculture Organization of the United Nations World Health Organization United Nations University . Human energy requirements: report of a joint FAO/WHO/UNU Expert Consultation. Food Nutr Bull. (2005) 26:166. 15810802

[22] Polonsky WH Fisher L Earles J Dudl RJ Lees J Mullan J . Assessing psychosocial distress in diabetes: development of the diabetes distress scale. Diabetes Care. (2005) 28:626–31. doi: 10.2337/diacare.28.3.626 15735199

[23] Ranjan R Rajput M Sachdeva A Saha A Jyotsana Yadav K . Prevalence of diabetes distress and cross-cultural reliability of DDS-17 scale in rural Haryana. J Fam Med Prim Care. (2023) 12:2064–9. doi: 10.4103/jfmpc.jfmpc_496_23 38024882 PMC10657097

[24] Phuwilert P Noo-In S Srichomphoo C Ruetrakul J Kongmant R Khiewkhern S . Effectiveness of a community-based health care program on glycemic control among patients with uncontrolled T2D mellitus: a quasi-experimental study. Diabetology. (2026) 7:14. doi: 10.3390/diabetology7010014 30654563

[25] Roth L Bretschneider MP Schwarz PEH . Randomized controlled trial to evaluate an app-based multimodal digital intervention for people with type 2 diabetes in comparison to a placebo app. Front Digit Health. (2025) 7:1644612. doi: 10.3389/fdgth.2025.1644612 41459242 PMC12738949

[26] Kulzer B Daenschel W Daenschel I Schramm W Messinger D Weissmann J . Integrated personalized diabetes management improves glycemic control in patients with insulin-treated type 2 diabetes: results of the PDM-ProValue study program. Diabetes Res Clin Pract. (2018) 144:200–12. doi: 10.1016/j.diabres.2018.09.002 30205184

[27] Lee SE Park SK Park YS Kim KA Choi HS Oh SW . Effects of short-term mobile application use on weight reduction for patients with type 2 diabetes. J Obes Metab Syndr. (2021) 30:345–53. doi: 10.7570/jomes21047 34875628 PMC8735826

[28] Pamungkas RA Usman AM Chamroonsawasdi K Abdurrasyid . A smartphone application of diabetes coaching intervention to prevent the onset of complications and to improve diabetes self-management: a randomized control trial. Diabetes Metab Syndr. (2022) 16:102537. doi: 10.1016/j.dsx.2022.102537 35724489

[29] Hilmarsdóttir E Sigurðardóttir ÁK Arnardóttir RH . A digital lifestyle program in outpatient treatment of type 2 diabetes: a randomized controlled study. J Diabetes Sci Technol. (2021) 15:1134–41. doi: 10.1177/1932296820942286 32680441 PMC8442170

[30] Tey J Lam J Yung CK Mulok MA Salleh NM Yong AML . Evaluation of the BALANCE program as a digital therapeutic solution for type 2 diabetes management: protocol for a prospective lifestyle intervention study. JMIR Res Protoc. (2025) 14:e73964. doi: 10.2196/73964 41359950 PMC12723359

[31] Kumari G Singh V Jhingan AK Chhajer B Dahiya S . Effectiveness of lifestyle modification counseling on glycemic control in patients with type 2 diabetes mellitus. Curr Res Nutr Food Sci J. (2018) 6:70–82. doi: 10.12944/CRNFSJ.6.1.07

[32] Rekha T Murali Mohan R Kumar N Hegde K Unnikrishnan B Mithra P . Obstacles for self-management practices among diabetes patients: a facility-based study from coastal South India. F1000Res. (2023) 12:839. doi: 10.12688/f1000research.138146.1 40115411 PMC11923534

[33] Kulkarni RK Sriram TR . Barriers to self-care behaviors among type 2 diabetes patients residing in rural and urban field practice areas of Belagavi, India. Natl J Community Med. (2025) 16:277–82. doi: 10.55489/njcm.160320254920

[34] Wangnoo SK Maji D Das AK Rao PV Moses A Sethi B . Barriers and solutions to diabetes management: an Indian perspective. Indian J Endocrinol Metab. (2013) 17:594–601. doi: 10.4103/2230-8210.113749 23961474 PMC3743358

[35] Green AJ Bazata DD Fox KM Grandy S SHIELD Study Group . Health-related behaviours of people with diabetes and those with cardiometabolic risk factors: results from SHIELD. Int J Clin Pract. (2007) 61:1791–7. doi: 10.1111/j.1742-1241.2007.01588.x 17887992 PMC2121151

[36] Holmen H Wahl AK Cvancarova Småstuen M Ribu L . Tailored communication within mobile apps for diabetes self-management: a systematic review. J Med Internet Res. (2017) 19:e227. doi: 10.2196/jmir.7045 28645890 PMC5501926

[37] Yang X Li Z Sun J . Effects of cognitive behavioral therapy-based intervention on improving glycaemic, psychological, and physiological outcomes in adult patients with diabetes mellitus: a meta-analysis of randomized controlled trials. Front Psychiatry. (2020) 11:711. doi: 10.3389/fpsyt.2020.00711 32848906 PMC7399630

[38] Lu X Yang H Ying X Lin S . Enhancing outcomes in type 2 diabetes: the role of psychological interventions within multidisciplinary rehabilitation care. BMC Nurs. (2025) 24:1221. doi: 10.1186/s12912-025-03877-1 41023663 PMC12482004

[39] Abbas Q Latif S Habib HA Shahzad S Sarwar U Shahzadi M . Cognitive behavior therapy for diabetes distress, depression, health anxiety, quality of life and treatment adherence among patients with type 2 diabetes mellitus: a randomized controlled trial. BMC Psychiatry. (2023) 23:86. doi: 10.1186/s12888-023-04546-w 36737757 PMC9896442

[40] Chapman A Liu S Merkouris S Enticott JC Yang H Browning CJ . Psychological interventions for the management of glycemic and psychological outcomes of type 2 diabetes mellitus in China: a systematic review and meta-analyses of randomized controlled trials. Front Public Health. (2015) 3:252. doi: 10.3389/fpubh.2015.00252 26636054 PMC4644788

[41] Goyal Mehra C Raymond AM Prabhu R . A personalized multi-interventional approach focusing on customized nutrition, progressive fitness, and lifestyle modification resulted in the reduction of HbA1c, fasting blood sugar and weight in type 2 diabetes: a retrospective study. BMC Endocr Disord. (2022) 22:290. doi: 10.1186/s12902-022-01212-2 36419152 PMC9685833

